# Association of Methylenetetrahydrofolate Reductase rs1801133 Polymorphism with osteoporosis and fracture risk in Taiwan

**DOI:** 10.7150/ijms.97524

**Published:** 2024-08-19

**Authors:** Meng-Hua Li, I-Chieh Chen, Hui-Wen Yang, Hsin-Chien Yen, Yu-Yuan Ke, Yi-Ming Chen, Chia-Chi Hsu

**Affiliations:** 1 Division of Pediatric Genetics and Metabolism, Children's Medical Center, Taichung Veterans General Hospital, Taichung, Taiwan.; 2 Department of Medical Research, Taichung Veterans General Hospital, Taichung, Taiwan.; 3 Department of Post-Baccalaureate Medicine, College of Medicine, National Chung Hsing University, Taichung, Taiwan.; 4 School of Medicine, National Yang Ming Chiao Tung University, Taipei, Taiwan.; 5 Division of Allergy, Immunology, and Rheumatology, Department of Internal Medicine, Taichung Veterans General Hospital, Taichung, Taiwan.; 6 Institute of Biomedical Science and Rong Hsing Research Center for Translational Medicine, National Chung Hsing University, Taiwan.

**Keywords:** osteoporosis, MTHFR, MTHFR rs1801133, fracture, bone mineral density

## Abstract

**Introduction:** Osteoporosis is a prevalent skeletal disorder influenced by age, hormonal changes, medication use, nutrition, and genetics. The relationship between MTHFR and osteoporosis remains unclear, especially in Asians. The aim of our study was to elucidate the impact of MTHFR on osteoporosis and fracture risk.

**Materials and Methods:** Participants were recruited from the Taiwan Precision Medicine Initiative at Taichung Veterans General Hospital. A total of 3,503 subjects with available bone mineral density measurements were selected. Using the Axiom Genome-Wide TWB 2.0 Array, we identified the MTHFR rs1801133 variant. Among these subjects, 1,624 patients carrying the variant were included in the case group, while the remaining 1,879 patients without the variant served as the control group.

**Results:** Overall, individuals carrying the MTHFR rs1801133 variant exhibited a significantly elevated risk of developing osteoporosis. Stratified analysis by different genotypes, the results revealed a statistically significant association between the heterozygous genotype of MTHFR rs1801133 and osteoporosis. However, there was no significant correlation between MTHFR genotypes and fracture risk. Furthermore, subgroup analysis of female patients revealed age, a known risk factor, was associated with both osteoporosis and fractures. Interestingly, the presence of the MTHFR rs1801133 variant did not confer an increased risk of osteoporosis or fractures in females.

**Conclusion:** Our study revealed a notable increase in the prevalence of osteoporosis among individuals carrying the MTHFR rs1801133 variant. Nevertheless, these individuals did not exhibit a heightened risk of major or hip fractures compared to non-carriers. Our findings could be of value in raising awareness of the increased risk of osteoporosis among individuals with this genetic variant.

## Introduction

Osteoporosis is a prevalent skeletal disorder characterized by the deterioration of bone quality and reduced bone mineral density, leading to osteoporotic fractures^1^. In the current aging population, osteoporosis poses a significant challenge, impacting both quality of life and mortality among the elderly. From a public health perspective, as life expectancy increases, osteoporosis also imposes a considerable socioeconomic burden^2^. Worldwide, the prevalence of osteoporosis is 19.7%, with a higher proportion seen among females (24.8%) compared to males (10.6%), and a particularly high prevalence in postmenopausal females (27.4%). Additionally, the prevalence varies across geographical regions, with higher rates observed in Asia and Africa than in Europe, America, and Oceania^3^.

**Figure 1:**
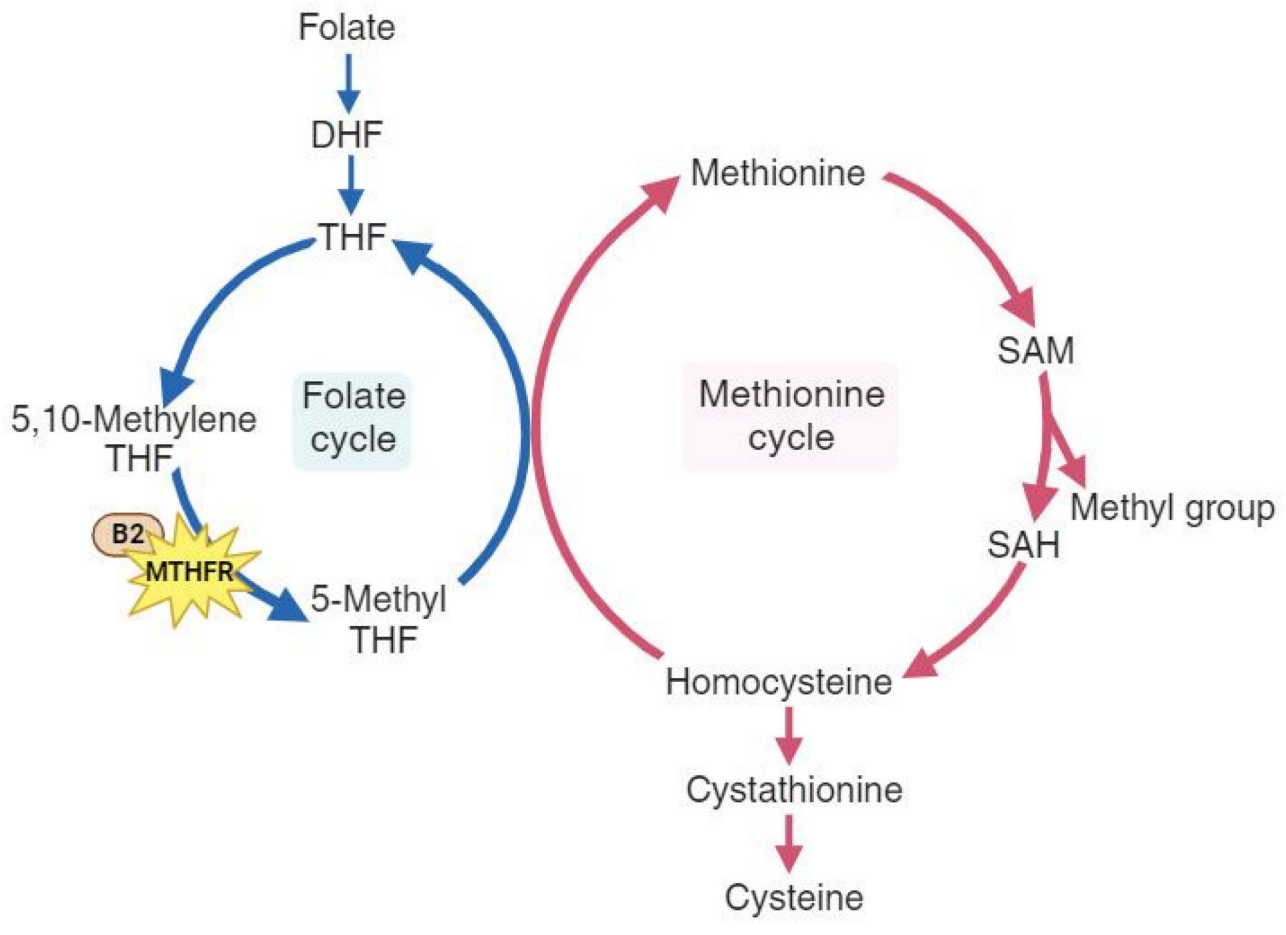
The pathways of folate metabolism (left cycle) and methionine metabolism (right cycle), highlighting the role of the MTHFR gene and the cofactor vitamin B2. DHF, dihydrofolate; THF, tetrahydrofolate; 5,10-methylene THF, 5,10-methylene tetrahydrofolate; 5-Methyl THF, 5-methyl tetrahydrofolate. B2, cobalamin (vitamin B2); SAM, S-adenosyl-methionine; SAH, S-adenosyl-homocysteine.

As a multifactorial disease, osteoporosis is influenced by age, hormonal changes, medication use, nutrition, and genetics^4^. Previous studies have shown that elderly individuals with hip bone loss and fractures often have elevated levels of homocysteine^5^, ^6^. Homocysteine metabolism is a crucial part of the methionine cycle, which is depicted in detail in Figure 1. During methionine degradation, homocysteine is formed through two steps involving the transfer of methyl groups, with intermediate production of S-adenosylmethionine (SAM) and S-adenosylhomocysteine (SAH). Subsequently, homocysteine is recycled back to methionine with a methyl group donated by 5-methyl tetrahydrofolate (5-MTHF). This 5-methyl THF is converted from 5,10-methylenetetrahydrofolate (5,10-MTHF) with the aid of the cofactor cobalamin and the enzyme methylenetetrahydrofolate reductase (MTHFR), which is encoded by the MTHFR gene.^7^, ^8^. A reduction in the enzyme methylenetetrahydrofolate reductase leads to elevated homocysteine levels and a decrease in methionine. Some studies have explored the impact of the MTHFR gene and have shown that dysfunction in MTHFR enzyme is associated with a higher risk of thromboembolic events, malignancy, major depression, and hypertension^9^-^12^. The relationship between MTHFR and osteoporosis has produced inconclusive results. The MTHFR rs1801133 variant (C677T) is a well-studied variant for its influence on osteoporosis and fractures. In Valero C *et al.*'s study^13^, this variant was not associated with osteoporotic fractures, while M. Shiraki *et al.*^14^ demonstrated that this variant may be a candidate for assessing fractures. Furthermore, in a meta-analysis conducted by H.-Z. Li *et al.*^15^, further evaluation in Asians was suggested to precisely establish the association between MTHFR and osteoporosis.

Therefore, based on prior studies, our objective was to validate the association between MTHFR and osteoporosis as well as fractures within the Taiwanese population. Additionally, we performed subgroup analyses to assess the effects of various genotypes, genders, and age groups. The data described herein shed light on the relationships among these variables.

## Material and Methods

### Study design and participants

This retrospective case-control study utilized data from the Taiwan Precision Medicine Initiative (TPMI). Between June 2019 and December 2021, all participants were recruited from Taichung Veterans General Hospital (TCVGH), and their electronic health records (EHR) were collected. The study population consisted of individuals aged 18 years and older with no prior medical history of cancer, all of whom provided written informed consent. EHR data collection took place between January 2009 and January 2022, involving approximately 58,091 volunteers. Among them, a total of 3,503 subjects with available bone mineral density (BMD) measurements were included in our analysis. The study protocol was approved by the Institutional Review Board of Taichung Veterans General Hospital (IRB No. SF19153A). Written informed consent was obtained from all participants in accordance with the principles defined in the Declaration of Helsinki.

### Genotyping and quality controls

**Figure 2:**
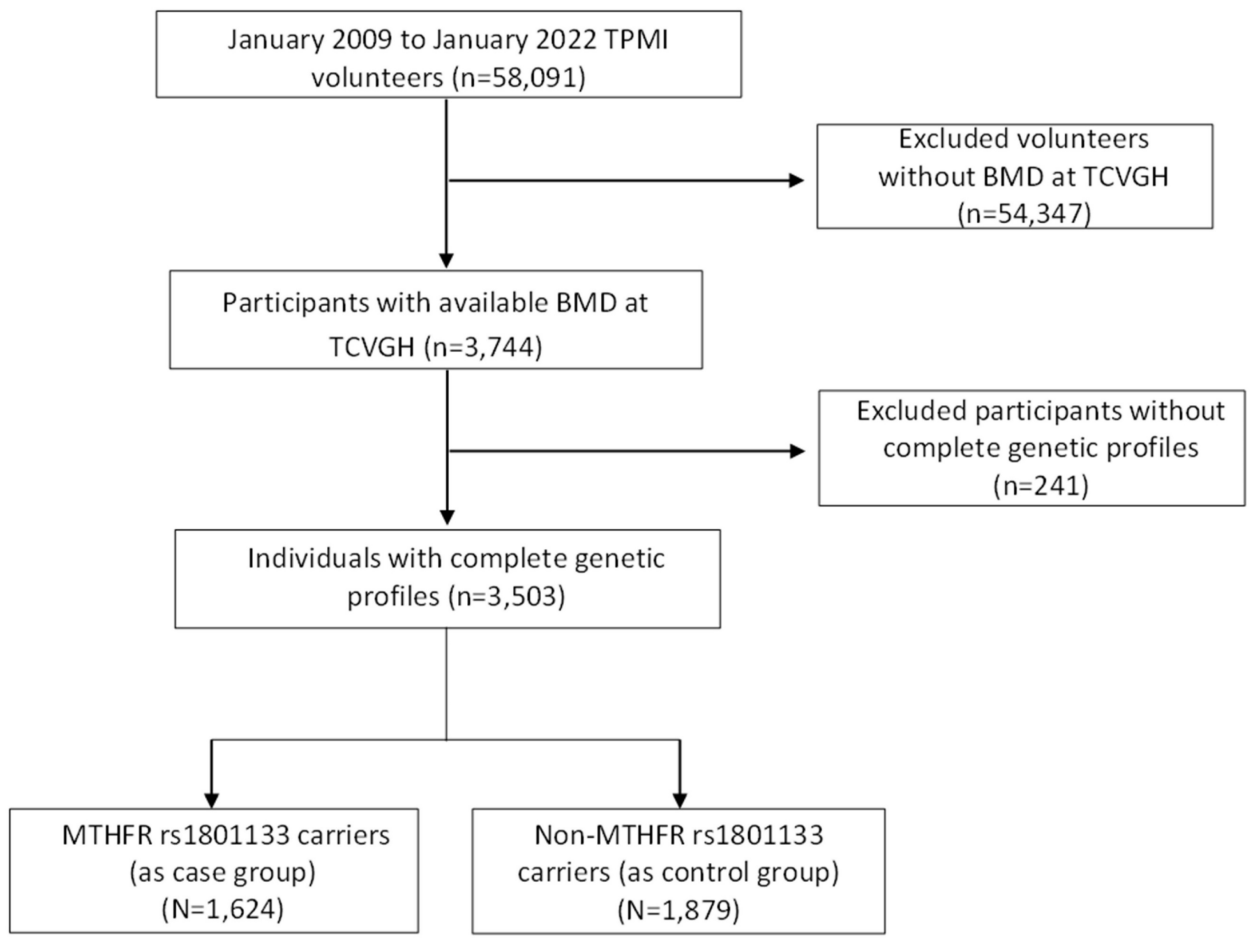
Study participants enrollment flow chart. TPMI, Taiwan Precision Medicine Initiative; BMD, Bone Mineral Density; TCVGH, Taichung Veterans General Hospital; MTHFR, Methylenetetrahydrofolate Reductase.

In this study, genomic DNA was isolated from 58,091 participants of the TPMI using DNA isolation kits sourced from TIANGEN Biotech, Beijing, China. Subsequently, the concentration of DNA samples was determined utilizing a NanoDrop 2000 Spectrophotometer (NanoDrop Technologies, Wilmington, DE, USA). Genotyping was done using the Axiom Genome-Wide TWB 2.0 Array Plate (Affymetrix, Santa Clara, CA, USA), which contains 714,431 SNPs and is designed specifically for Taiwan's Han Chinese population^16^. The use of high coverage SNP data from large-scale Han Chinese ancestry in Taiwan using custom arrays has been previously described^17^. Genotyping quality control procedures were implemented, which involved the exclusion of single-nucleotide polymorphisms (SNPs) displaying a sole allele occurrence within the study cohort, as well as those with a total call rate below 95% or a total minor allele frequency (MAF) below 0.01. Furthermore, SNPs exhibiting significant deviations from the Hardy-Weinberg equilibrium (P < 1 × 10^-5^) were also excluded. For statistical genetic analyses and genetic data quality control, the PLINK 1.9 software package was employed. Then, we selected 3503 participants for whom genotyping information for MTHFR rs1801133 was available. This polymorphism involves a single base mutation from G to A, resulting in an amino acid change from Alanine (A) to Valine (V). Genotyping of this polymorphism was categorized into three groups: GG representing the wild type, GA representing heterozygous carriers, and AA representing homozygous mutants. Among the total of 3505 participants, 1624 individuals were identified as carriers of the MTHFR rs1801133 polymorphism with at least one A allele mutation, forming the case group. The remaining 1879 participants were classified as wild type and thus served as the control group. The participant enrollment process is illustrated in Figure 2.

### Definition of covariates

In this study, the identification and diagnosis of various comorbidities were based on the International Classification of Diseases, Ninth Revision (ICD-9), and Tenth Revision (ICD-10), Clinical Modification diagnostic codes, including stroke (ICD-9-CM code 434.91, ICD-10-CM code I63.9), dementia (ICD-9-CM code 290, ICD-10-CM code F03.90 and F84.3), deep vein thrombosis (DVT) (ICD-9-CM code 453.8, ICD-10-CM code I82.409), pulmonary embolism(PE) (ICD-9-CM code 415.19, ICD-10-CM code I26.99), coronary artery disease(CAD) (ICD-9-CM code 414.9, ICD-10-CM code I25.9), diabetes mellitus (DM) (ICD-9-CM code 250, ICD-10-CM code E11.9), hypertension (ICD-9-CM code 401-405, ICD-10-CM code I10-I15), dyslipidemia (ICD-9-CM code 272.8, ICD-10-CM code E78.5), osteoporosis (ICD-9-CM code 733, ICD-10-CM code M81.0), major depressive disorder (ICD-9-CM code 296.2, ICD-10-CM code F32.9), schizophrenia (ICD-9-CM code 295.9, ICD-10-CM code F20.9 and F84.5), and hypothyroidism (ICD-9-CM code 244.9, ICD-10-CM code E03.9 and P72.2). We defined a major fracture as an individual hospitalized with diagnostic codes (ICD-9 codes 733, 805, 813.4, 820, and ICD-10 codes M80, S22, S32, S52, S72) or those who had a hip fracture. The definition of a hip fracture was based on a hospitalized patient undergoing hip fracture repair with the surgery codes 33126B, 33127B, 64029B, or 64170B. These surgery codes were determined according to the reimbursement codes provided by Taiwan's Bureau of National Health Insurance.

Laboratory data included lipid profiles (total cholesterol, low-density lipoprotein [LDL], high density lipoprotein [HDL], and triglyceride [TG]), thromboembolic parameter (Platelet [PLT], D-dimer, Protein C, Protein S, Antithrombin III), blood sugar status (hemoglobin A1c [HbA1c] and fasting glucose level), thyroid profile (thyroid stimulating hormone [TSH], Free T4, T4, T3), serum creatinine, calcium, phosphate, alkaline phosphatase (Alk-P) and 25(OH)D3 level, folate profile (hemoglobin [Hb], mean corpuscular volume [MCV] and folic acid). Logistic regression was used to analyze the associations between MTHFR variant and the collected laboratory data.

The evaluation of osteoporosis included conducting a bone mineral density (BMD) test and utilizing fracture risk assessment tools such as FRAX-major score and FRAX-hip score. Osteoporosis was defined as a T-score ≤ -2.5, in accordance with the criteria outlined by the World Health Organization (WHO)^18^. Bone mineral density (BMD) assessments of bilateral femoral necks and the lumbar spine (L1-L4) were conducted using dual-energy X-ray absorptiometry (DXA) with the Lunar Prodigy system (General Electric, Fairfield, CT, USA). The analysis categorized BMD scores into three sites: BMD-spine, BMD-left hip, and BMD-right hip. BMD was measured in grams per square centimeter (g/cm²), and T-scores were utilized. The BMD test encompassed evaluations of the spine and bilateral hip joints, with scores derived from measurements of bone mineral density and T-score calculations. DXA was employed to obtain BMD results, which were expressed in g/cm², and the T-score was defined as the standard deviation of an individual's BMD compared to that of healthy young adults.

A Taiwan-specific FRAX model was employed to calculate the 10-year probability of major osteoporotic fracture risk and the 10-year probability of hip fracture, with the inclusion of BMD measurement^19^. Therapeutic thresholds were defined as a 10-year probability of major osteoporotic fracture risk exceeding 20% and a 10-year probability of hip fracture surpassing 3%. This information was documented in the patient's medical records system.

### Statistical analysis

The statistical analyses were conducted using the SAS version 9.4 (SAS Institute Inc., Cary, NC). Statistical significance was defined as p-values less than 0.05. Categorical variables were presented as number (percent), while mean ± standard deviation (SD) was used for continuous data. Categorical variables were analyzed by the Chi-squared test. In cases where the minimum expected count was less than 5, Fisher's exact test was utilized. Continuous variables were examined using Student's t-test. Subgroup analyses were conducted based on gender and age divided into two groups, <55 years and ≥55 years. The odds ratios (OR) and 95% confidence intervals (95% CI) of the MTHFR rs1801133 variant were calculated using univariable and multivariable logistic regression analysis to assess the effects of the variant on osteoporosis and fracture. Adjustments were made for quantitative variables.

## Results

### Basic demographic analysis with MTHFR rs1801133

In our study, we analyzed data from 3,503 participants with available bone mineral density (BMD) measurements obtained from the EHR of TPMI. These volunteers underwent dual-energy X-ray absorptiometry (DXA) to assess BMD and T-scores. Among the 3,503 participants, 1,624 individuals (46.4%) were identified as carrying the MTHFR rs1801133 variant and constituted the case group, while 1,879 individuals (53.6%) were non-MTHFR carriers and formed the control group.

Table 1 presents the basic demographic characteristics of the study population. Nearly three-quarters of the participants were female. Regarding the MTHFR rs1801133 polymorphism, our study revealed a significant association between the variant and the risk of osteoporosis compared to those without the variant (53.63% vs. 49.39%, p=0.0122). The MTHFR rs1801133 variant did not show a significant association with the risk of fractures, including major fractures and hip fractures. Moreover, the analyses of the risk of other comorbidities such as dementia, thromboembolic events (deep vein thrombosis, pulmonary embolism, coronary artery disease, and acute myocardial infarction), metabolic diseases (diabetes mellitus, hypertension, dyslipidemia, and hypothyroidism), and psychological diseases (major depressive disorder and schizophrenia) revealed no statistical significance.

The biochemistry data of the study population are illustrated in Table 2. Individuals with the MTHFR rs1801133 variant demonstrated lower levels of total cholesterol (192.68 ± 43.31 vs. 195.9 ± 42.58 mg/dL, p = 0.0428), which was statistically significant. Nonetheless, the remaining results showed similarities between the two groups and did not reach statistical significance.

### Osteoporosis-related serology and evaluation of study population

Table 3 illustrates the correlation between osteoporosis and the MTHFR rs1801133 variant. Osteoporosis-related serology included serum creatinine, calcium, phosphate, alkaline phosphatase (Alk-P), and 25(OH)D3 levels. Bone mineral density and T-scores were utilized to evaluate the severity of bone loss. The results of analyses comparing the effects of the MTHFR rs1801133 variant revealed that in participants carrying the variant, the BMD of the right hip joint measured 0.76 g/cm^2^, which was lower than that of non-carriers(1.07 g/cm^2^); however, no significant association was noted. Elsewhere, minimal differences in serology and BMD score were observed between the two groups, with no statistical significance.

As for the incidence of osteoporosis and fracture, the variant was associated with a significantly higher risk of developing osteoporosis (53.63% vs. 49.39%, p=0.0122), as previously shown in Table 1. However, there was no statistically significant difference in the prevalence of major and hip fractures, nor in the 10-year fracture probability, presented as FRAX major score and FRAX hip score.

### Factors associated with osteoporosis susceptibility

As demonstrated in Table 4, univariable logistic regression was conducted to investigate specific risk factors related to osteoporosis. The risk of osteoporosis was significantly associated with age ≥ 55 years old (OR: 5.066; 95% CI: 4.074-6.4, p<0.0001), female gender (OR: 1.75; 95% CI: 1.497-2.045, p<0.0001) and MTHFR rs1801133 GA genotype (OR: 1.17; 95% CI: 1.018-1.346, p=0.0275) respectively.

In addition to the elevated incidence of osteoporosis development, we also examined the risk factors for suffering major and hip fractures using multivariable logistic regression analysis, with adjustment for age, sex, and FRAX. As depicted in Table 5, it is evident that individuals aged ≥ 55 years (OR: 6.361; 95% CI: 3.255-12.431, p<0.0001) had a higher risk of major fractures, as did those with FRAX-major scores exceeding 20% (OR: 1.057; 95% CI: 1.042-1.071, p<0.0001). Conversely, in participants with the MTHFR rs1801133 genotype, there was no relationship with major fractures. Moreover, gender did not appear to influence the risk of major fractures.

Furthermore, individuals aged ≥ 55 years (OR: 8.075; 95% CI: 1.981-32.917, p=0.0036) and those with FRAX-hip scores exceeding 3% (OR: 1.077; 95% CI: 1.051-1.104, p<0.0001) had an increased risk of hip fractures. Interestingly, female gender (OR: 0.635; 95% CI: 0.403-0.999, p=0.0495) demonstrated a protective effect against hip fractures. As for the variant, no significant association was observed between MTHFR rs1801133 genotype and hip fractures. These results indicated that age is a significant factor associated with major fractures and hip fracture.

### Factor and osteoporosis in females

In a subgroup analysis presented in Table 6, we further stratified participants according to gender and age. Univariable logistic regression revealed that in females, there was a remarkable association between osteoporosis and age ≥ 55 years (OR: 5.537; 95% CI: 4.35-7.048, p<0.0001). The effect of the MTHFR rs1801133 variant on osteoporosis did not achieve statistical significance in females. In Table 6, of particular note, when divided by age, regardless of whether the age was below or ≥ 55 years, age remains significantly associated with osteoporosis. Similarly, compared to non-carriers irrespective of age, no statistically significant impacts of the MTHFR rs1801133 genotypes on osteoporosis were found.

In the development of fractures in females, as shown in Table 7, age consistently emerged as the crucial factor. Specifically, individuals aged ≥ 55 years exhibited significantly higher risk, with or without major fracture (OR: 5.011; 95% CI: 2.55-9.844, p<0.0001) or hip fracture (OR: 5.568; 95% CI: 1.353-22.906, p = 0.0174). For female participants, FRAX-major score exceeding 20% (OR: 1.069; 95% CI: 1.051-1.086, p<0.0001) and FRAX-hip score exceeding 3% (OR: 1.09; 95% CI: 1.059-1.121, p<0.0001) were significantly correlated to bone injuries including major fractures and hip fractures. In contrast, the association of MTHFR rs1801133 GA and AA genotypes with fracture did not attain statistical significance for either fracture type.

## Discussion

We explored the impact of MTHFR polymorphism on osteoporosis and fracture events. Individuals carrying the MTHFR rs1801133 variant exhibited a significantly higher incidence of osteoporosis. Overall, age, female gender, and the MTHFR rs1801133 variant were identified as factors associated with osteoporosis. In terms of the development of major and hip fractures, individuals who were elderly, female, or had elevated FRAX scores showed a significantly increased prevalence. In females, we found that age was a significant factor associated with osteoporosis and bone injuries, such as major and hip fractures. Similarly, females with higher FRAX scores also showed an increased risk of fractures. Although statistical significance was not reached, we identified a potential trend between the MTHFR rs1801133 variant and osteoporosis in females, irrespective of age. In addition, females who were heterozygous carriers of the MTHFR rs1801133 variant were not more prone to major and hip fractures compared to the control group, although this was non-significant. Our study aimed to elucidate the potential trend of MTHFR heterozygous carriers in developing osteoporosis and fractures. While numerous studies have investigated the impact of MTHFR, to the best of our knowledge, ours is the first to examine the association between MTHFR and osteoporosis within the Taiwanese population.

In MTHFR polymorphisms, it has been observed that the MTHFR mutation is associated not only with osteoporosis and fractures but also with a range of other conditions, including thromboembolic events, malignancies, psychiatric disorders, hypertension, cognitive impairment and Alzheimer's disease, etc^9^-^12^, ^20^. The reduction of the enzyme methylenetetrahydrofolate reductase results in elevated total plasma homocysteine levels and reduced methionine levels. In a study by Grossi *et al.*^21^, Alzheimer's disease was associated with elevated homocysteine levels and reduced levels of cobalamin and folate. Additionally, Román *et al.*^20^ reported that elevated homocysteine contributes to oxidative stress, leading to a decrease in SAM levels. This, consecutively, induces DNA demethylation, which triggers the overexpression of Alzheimer's disease-related genes through epigenetic changes. The resulting neuronal dysfunction leads to problems with learning, memory, and behavior, ultimately contributing to the development of late-onset Alzheimer's disease (LOAD). Moreover, Tang *et al.*^22^ identified a synergistic effect of decreased folate levels and the MTHFR C677T mutation, leading to hippocampal subregions atrophy in individuals with Alzheimer's disease. The structural alterations associated with the genetic effects of MTHFR C677T mutation have also been documented additionally. A significant reduction in the volume of the right precuneus was observed in individuals with the MTHFR T variant compared to those with the wild type, regardless of whether they were in the healthy control group or the amnestic mild cognitive impairment (aMCI) group^23^. The precuneus, located in the parietal lobe, is responsible for functions such as visuo-spatial imagery, episodic memory retrieval, and self-processing^24^. While the link between MTHFR and Alzheimer's disease is well-documented, the association between MTHFR polymorphisms and cognitive impairment remains less clear. For instance, Sun *et al.*^25^ found that the MTHFR rs1801133 variant was not associated with susceptibility to mild cognitive impairment. Additionally, although individuals homozygous for the MTHFR rs1801133 variant exhibited higher homocysteine levels in Parkinson's disease (PD) patients, no significant correlation was found between MTHFR polymorphisms and cognitive impairment in these patients^26^.

The impact of MTHFR polymorphism on osteoporosis and fractures has been extensively studied, initially yielding conflicting results. Li *et al.*^27^ conducted an analysis demonstrating no association between MTHFR and BMD in either Chinese men or women. In contrast, a study by Bathum *et al.*^28^ revealed that individuals with either heterozygous or homozygous MTHFR variants exhibited a significantly increased risk of fractures. As the findings in the literature appeared to be inconclusive, several authors conducted meta-analyses. Wang *et al.*^29^ reported that the MTHFR variant exhibited marginal evidence of increased fracture risk, particularly in East Asians, females, and individuals aged below 60 years, though these results were not significant. Furthermore, some meta-analyses focused on the impact of MTHFR on the risk of osteoporosis and BMD. DH Li *et al.*^30^ reported that the MTHFR variant in postmenopausal women was linked to lower femoral neck BMD, with the TT genotype considered a significant risk factor for postmenopausal osteoporosis. Additionally, HZ. Li *et al.*^15^ established an association between the MTHFR variant and reduced BMD in the lumbar spine and femoral neck, particularly in Caucasians and postmenopausal women. It is noteworthy that in a study by HZ Li et al., no association between the MTHFR variant and BMD was observed in the Asian subgroup due to data heterogeneity. Further investigation in Asians was suggested by HZ Li. Our study provides a more comprehensive evaluation of this relationship among Asians. We demonstrated that the BMD of the spine and bilateral hips in MTHFR carriers was lower compared to that of non-carriers, although no significant association was observed. However, we did establish a significant correlation between the MTHFR polymorphism and osteoporosis. We speculate that clinicians might have diagnosed osteoporosis using broader criteria. As reported by Siris *et al.*^31^, osteoporosis diagnosis could be applied to individuals at elevated risk of fracture or those who have experienced hip fracture episodes, regardless of BMD results. The results of some Asian studies are consistent with these findings. In a study by Sanjeev Pandey *et al.*^32^, among premenopausal north Indian women, those with the TT genotype of the MTHFR gene exhibited a significant decrease in BMD at the lumbar spine, femur, and forearm compared to those with the CC genotype. The study was also included in the meta-analysis conducted by HZ Li *et al.* In a 2013 study, Tongboonchoo C *et al.*^33^ reported that postmenopausal Thai women with the MTHFR C677T heterozygous genotype had a significant risk of osteopenic bones compared to normal controls. In contrast, a 2018 study by Ahn *et al.*^34^ demonstrated MTHFR 2572C>A had a statistically significant protective effect against osteoporosis in Korean postmenopausal women. Though diverse variants of the MTHFR gene have been investigated, the clinical impact of the MTHFR gene on osteoporosis has still been inconsistent.

As demonstrated in our study, the risk of fracture was not solely attributed to osteoporosis. We found that female carriers were at risk of developing osteoporosis, but there was no increased potential for fracture development. We hypothesize that this variation could be due to the age, which may explain the absence of fractures. Several studies have explored the relationship between osteoporosis and fracture risk. For instance, in a study by HL Li *et al.*^35^, it was observed that the fracture risk increased by 1.6 to 1.7 times for every one-point reduction in T-score (BMD). In terms of fractures, factors beyond BMD should also be considered. For individuals without osteoporosis, both men and women with postural instability or falling history should be identified, as they are at risk of fractures even before developing osteoporosis^36^. As for postmenopausal women, in addition to a history of fractures or falls, factors such as age, cognitive function, and overall health condition are of importance in determining the risk of imminent fractures^37^. The evaluation of fracture risk should be feasible even with identical BMD in individuals of different ages. In the aging process, the elderly are at a greater risk of fracture compared to younger individuals, because the former tend to experience skeletal loss and reduced bone strength^38^. Therefore, fall prevention, avoidance of muscle weakness, and maintaining general health are also important. There are several parameters involved in assessing the risk of fractures. In the current study, we determined that regardless of the MTHFR genotype, the risk of fractures could not be independently accounted for. In addition, regarding major and hip fractures, we found no significant association between MTHFR rs1801133 carriers and non-carriers.

In the subgroup analysis, we specifically explored menopausal females. As the age at menopause was not necessarily available in medical records, we defined the age of 55 years as the threshold for menopause. This decision was guided by the findings of T.-Y. Shen *et al.*^39^, who reported a mean age at menopause of 50.2 years with a standard deviation of 4.0 years, in a cohort analysis in Taiwan. For menopausal females with the MTHFR variant, our study revealed that heterozygous carriers showed no significant association with osteoporosis development compared to GG genotype subjects, nor did AA genotype homozygous females. The A allele did not appear to increase the risk of osteoporosis. A similar finding was reported in a study by H.L. Jorgensen *et al.*^40^, which found that distal forearm BMD did not vary significantly across different MTHFR genotypes in Danish postmenopausal women. Furthermore, the study by H.L. Jorgensen *et al.* showed that in the wild type, women with the homozygous G allele had a significantly increased odds ratio of fractures. However, a study by M. Miyao *et al.*^41^ demonstrated a significant association of the MTHFR variant with lower lumbar and total BMD in postmenopausal Japanese women, although this study primarily focused on the homozygous AA genotype rather than the heterozygous genotype. We postulate that the inconsistent results may be attributed to different allele frequencies among various ethnic groups and geographic regions^42^. Higher frequencies were observed in Hispanic (42%) and Chinese populations (42%), followed by Caucasians (32%), with the lowest frequency found in African-Americans (16%)^43^, ^44^. Further research in different population is needed to explore genetic susceptibility factors in osteoporosis.

In this study, there were some limitations. First, osteoporosis is a polygenic disease, and the MTHFR gene's effect may be one of the crucial modulators in bone metabolism. Prediction models for low BMD with osteoporotic fracture have been established using a polygenic score approach 45-47. Additionally, other potential confounders such as physical activity, sleep duration^48^, family history of fracture, and steroid use^1^ were not recorded in the study. Second, the BMD scores in our study were based on the participants' initial medical records, which may have underestimated the prevalence of osteoporosis if subsequent BMD scores were lower. Third, the BMD examination relied on the participants' clinical conditions as assessed by physicians, and thus selection bias due to the limited sample size in this hospital-based case-control study may have existed. Additional longer-term prospective investigations of the associations of MTHFR with osteoporosis and fracture are needed to clarify these issues.

## Conclusion

We analyzed the relationship between the MTHFR variant and osteoporosis as well as fractures, marking the first such report within the Taiwanese population. Our finding indicated that individuals with the MTHFR rs1801133 variant had a significantly higher prevalence of osteoporosis. However, the risk of developing major and hip fractures was not greater than that of non-carriers. Age and gender continue to be a significant factor associated with osteoporosis and fractures. These findings contribute to a comprehensive understanding of the impact of MTHFR variants on osteoporosis and fractures in the Taiwanese population. From the perspective of disease prevention, our findings could be utilized to enhance awareness about osteoporosis among individuals with this variant and to implement osteoporosis screening programs more effectively.

## Figures and Tables

**Table 1:** Basic characteristics of the study population.

Variables	MTHFR rs1801133 carriers (N=1624)			Non-carriers (n=1879)		*P* value
	Mean	SD		Mean	SD	
**Age^a^**	67.42	13.16		67.11	12.89	0.4859
**BMI^a^**	24	3.99		23.89	3.93	0.539
	**N**	**%**		**N**	**%**	
**Gender^b^**						0.5868
Female	1217	74.94		1423	75.73	
Male	407	25.06		456	24.27	
**Comorbidities^b^**						
Dementia	63	3.88		69	3.67	0.7482
Stroke	112	6.9		121	6.44	0.5883
Deep vein thrombosis	36	2.22		38	2.02	0.6899
Pulmonary embolism	11	0.68		22	1.17	0.1316
CAD (coronary artery disease)	80	4.93		79	4.2	0.3061
DM (diabetes mellitus)	457	28.14		532	28.31	0.9099
Hypertension	3	0.18		7	0.37	^c^0.3560
Dyslipidemia	37	2.28		45	2.39	0.82
Hypothyroidism	115	7.08		157	8.36	0.1599
Osteoporosis	871	53.63		928	49.39	0.0122
Major fracture	128	7.88		162	8.62	0.4281
Hip fracture	38	2.34		48	2.55	0.6823
Major Depressive Disorder	0	0		0	0	-
Schizophrenia	1	0.06		1	0.05	^c^1.00

^a^Continuous variables were expressed as mean ± standard deviation (SD) and were analyzed using Student's t-test for normal data distributions ^b^Categorical variables were expressed as numbers (percent) and were analyzed using the Chi-square test. ^c^Categorical variables were expressed as numbers (percent) and were analyzed using the Fisher's exact probability test.

**Table 2:** Basic biochemistry characteristics of the study population.

Variables	MTHFR rs1801133 carriers (N=1624)			Non-carriers (n=1879)		*P* value
	Mean	SD		Mean	SD	
**Biochemistry (Mean/SD)^a^**						
Total cholesterol (mg/dL)	192.68	43.31		195.9	45.58	0.0428
HDL (mg/dL)	60.5	18.11		59.64	17.67	0.3039
LDL (mg/dL)	115.01	36.69		116	37.43	0.479
Triglyceride (mg/dL)	127.69	98.44		133.06	124.45	0.1771
Platelet (x10^3/uL)	246.12	75.4		244.32	81.04	0.5011
D-dimer (mg/l FEU)	22.58	93.21		39.25	156.44	0.0543
Protein C (%)	111.2	19.59		112	39.3	0.9549
Protein S (%)	76.6	13.21		89.6	24.62	0.3148
Antithrombin III (%)	94.83	15.73		74.33	22.37	0.1489
HbA1c (%)	6.24	1.51		6.26	1.48	0.6683
Fasting glucose (mg/dL)	108.96	40.34		110.21	35.26	0.3995
TSH (uIU/mL)	2.98	12.16		3.03	13.22	0.9283
Free T4 (ng/dL)	9.62	6.52		9.66	5.83	0.8765
T4 (ug/dL)	9.23	21.74		7.42	2.06	0.4004
T3 (ng/dL)	81.11	42.5		84.43	57.8	0.4956
Alk-p (U/L)	96.89	58.87		97.93	69.21	0.6903
Hb (g/dL)	12.74	1.8		12.75	1.89	0.856
MCV (fL)	90.14	7.81		89.78	7.92	0.1968
Folic acid (ng/mL)	13.49	7.83		14.25	8.21	0.2521

^a^Continuous variables were expressed as mean ± standard deviation (SD) and were analyzed using Student's t-test for normal data distributions

**Table 3:** Osteoporosis-related measurement of the study population.

Variables	MTHFR rs1801133 carriers (N=1624)			Non-carriers (n=1879)		*P* value
	Mean	SD		Mean	SD	
**Lab test**						
Creatinine (mg/dL)^a^	1.43	2.25		1.48	3.2	0.6185
Calcium (mg/dL)^a^	9.02	0.61		9.05	0.62	0.248
Phosphate (mg/dL)^a^	3.86	1.19		3.82	1.13	0.3708
25(OH)D3 (mg/dL)^a^	29.35	14.26		29.57	12.08	0.867
**BMD score**						
BMD, Spine (g/cm2)^a^	1.03	0.31		1.65	26.22	0.3166
BMD, left hip (g/cm2)^a^	0.77	0.13		0.77	0.14	0.609
BMD, right hip (g/cm2)^a^	0.76	0.13		1.07	12.75	0.3134
T-score, spine^a^	-1.04	1.67		-0.94	1.72	0.1139
T-score, left hip^a^	-1.73	1.08		-1.69	1.08	0.2737
T-score, right hip^a^	-1.75	1.05		-1.71	1.04	0.3014
**Osteoporosis and fracture**						
FRAX-major (%)^a^	12.93	10.34		12.64	9.42	0.5772
FRAX-hip (%) ^a^	5.5	6.55		5.46	6.61	0.9079
	**N**	**%**		**N**	**%**	
Osteoporosis	871	53.63		928	49.39	0.0122
Major Fracture	128	7.88		162	8.62	0.4281
Hip Fracture	38	2.34		48	2.55	0.6823

^a^Mean ± SD; Abbreviation: BMD: Bone mineral density; FRAX: the Fracture Risk Assessment Tool; FRAX-major: 10-year probabilities of major osteoporotic fracture; FRAX-hip: 10-year probabilities of hip fracture.

**Table 4:** Factors associated with osteoporosis of the study population.

Osteoporosis	OR	95% CI	*P* value^a^
**Age**			
<55	1		
>=55	5.066	4.074-6.3	<0.0001
**Sex**			
Male	1		
Female	1.75	1.497-2.045	<0.0001
**MTHFR rs1801133**			
GG	1		
GA	1.17	1.018-1.346	0.0275
AA	1.266	0.978-1.639	0.0733

^a^ORs were estimated using univariable logistic regression.

**Table 5:** Factors associated with major and hip fractures of the study population.

**Major fracture**	**OR**	**95% CI**	***P* value^c^**
**Age**			
<55	1		
>=55	6.361	3.255-12.431	<0.0001
**Sex**			
Male	1		
Female	1.009	0.763-1.334	<0.9497
**FRAX-major^a^**	1.057	1.042-1.071	<0.0001
**MTHFR rs1801133**			
GG	1		
GA	0.852	0.657-1.103	0.2241
AA	1.198	0.78-1.84	0.4104
**Hip fracture**	**OR**	**95% CI**	***P* value^c^**
**Age**			
<55	1		
>=55	8.075	1.981-32.917	0.0036
**Sex**			
Male	1		
Female	0.635	0.403-0.999	0.0495
**FRAX-hip^b^**	1.077	1.051-1.104	<0.0001
**MTHFR rs1801133**			
GG	1		
GA	0.891	0.564-1.408	0.6211
AA	1.031	0.462-2.303	0.9407

^a^FRAX-major risk exceeding 20% vs. FRAX-major risk below 20%. ^b^FRAX-hip risk exceeding 3% vs. FRAX-hip risk below 3%. ^c^By logistic regression with adjustment of age, sex, and FRAX.

**Table 6:** Factors associated with osteoporosis in females and subgroups.

**Osteoporosis in females**	**OR**	**95% CI**		***P* value^a^**
**Age**	1.08	1.072-1.089		<0.0001
**Sex, female**				
<55	1			
>=55	5.537	4.35-7.048		<0.0001
**MTHFR rs1801133**				
GG	1			
GA	1.069	0.909-1.256		0.4216
AA	1.227	0.908-1.658		0.1822
**Osteoporosis, females, age <55**	**OR**	**95% CI**		***P* value^a^**
**Age**	1.041	1.002-1.083		0.0416
**MTHFR rs1801133**				
GG	1			
GA	1.352	0.841-2.174		0.2127
AA	1.869	0.799-4.374		0.1491
**Osteoporosis, females, age >=55**	**OR**	**95% CI**		***P* value^a^**
**Age**	1.082	1.017-1.094		<0.0001
**MTHFR rs1801133**				
GG	1			
GA	1.043	0.871-1.25		0.6468
AA	1.131	0.811-1.579		0.4678
				

^a^ORs were estimated using univariable logistic regression.

**Table 7:** Factors associated with major and hip fractures in females.

**Major fracture**	**OR**	**95% CI**	***P* value^c^**
**Age**	1.066	1.052-1.08	<0.0001
**Sex, female**			
<55	1		
>=55	5.011	2.55-9.844	<0.0001
**FRAX-major^a^**	1.069	1.051-1.086	<0.0001
**MTHFR rs1801133**			
GG	1		
GA	0.821	0.608-1.108	0.198
AA	1.236	0.759-2.015	0.3943
**Hip fracture**	**OR**	**95% CI**	***P* value^c^**
**Age**	1.084	1.057-1.111	<0.0001
**Sex, female**			
<55	1		
>=55	5.568	1.353-22.906	0.0174
**FRAX-hip^b^**	1.09	1.059-1.121	<0.0001
**MTHFR rs1801133**			
GG	1		
GA	0.754	0.429-1.326	0.3274
AA	0.607	0.185-1.992	0.4104

^a^FRAX-major risk exceeding 20% vs. FRAX-major risk below 20%. ^b^FRAX-hip risk exceeding 3% vs. FRAX-hip risk below 3%. ^c^By logistic regression with adjustment of age, sex, and FRAX.
